# From Migration to Emergency: A Systematic Review of Acute Presentations Following Late Esophageal Stent Migration

**DOI:** 10.3390/jcm15135092

**Published:** 2026-06-30

**Authors:** Adam Mylonakis, Konstantina Felekoura, Michail-Panagiotis Pontikas, Spyros I. Siakavellas, Chrysovalantis Vergadis, Dimosthenis Chrysikos, Andreas Koutsoumpas, Dimitrios Schizas

**Affiliations:** 1 First Department, Surgery, National and Kapodistrian University of Athens, Laikon General Hospital, 11527 Athens, Greece; kon.felekoura@gmail.com (K.F.); mixalispnt@gmail.com (M.-P.P.); schizasad@gmail.com (D.S.); 2 Hepatogastroenterology Unit, Second Department, Internal Medicine, National and Kapodistrian University of Athens, Hippocration General Hospital, 11527 Athens, Greece; s.siakavellas@gmail.com; 3 Third Department, Radiology, National and Kapodistrian University of Athens, Laikon General Hospital, 11527 Athens, Greece; valvergadis@gmail.com; 4 Department of Anatomy, School of Medicine, National and Kapodistrian University of Athens, 11527 Athens, Greece; dixrys@yahoo.gr; 5 First Department, Gastroenterology, National and Kapodistrian University of Athens, Laikon General Hospital, 11527 Athens, Greece; andreas.koutsoumpas@laiko.gr

**Keywords:** esophageal stent migration, self-expanding metal stent, stent complications

## Abstract

**Background**: Self-expanding esophageal stents (SEMS) are widely used in the management of malignant dysphagia and a variety of benign esophageal conditions. Although stent migration is a well-recognized complication, late migration -defined as occurring ≥4 weeks after stent placement- is less well characterized and may result in severe, life-threatening emergencies. These events are frequently underrecognized and not consistently addressed within current surveillance practice. **Methods**: A systematic review was conducted in PubMed/MEDLINE, Embase, and Scopus databases focusing on acute clinical presentations following late esophageal stent migration. **Results**: Out of 343 unique articles, 53 studies were included, involving 53 patients with a median time to presentation of 3 months after stent placement, and 12.5% of cases occurring more than one year later. The gastrointestinal tract was the most frequent site of migration (64.2%), followed by airway/respiratory (15%), thoracic (13.2%), and vascular structures (5.7%). Clinical presentations included small bowel obstruction (30.2%), gastrointestinal or thoracic perforation, respiratory failure, sepsis, and catastrophic hemorrhage. Surgical intervention was required in most cases. Overall mortality was 28.3%, with especially poor outcomes in cases complicated by vascular or airway involvement. **Conclusions**: Late esophageal stent migration represents a clinically significant but underrecognized cause of acute surgical emergencies. Risk-adapted surveillance, prompt diagnostic imaging, and early multidisciplinary management are essential to improve outcomes and enhance the long-term safety of esophageal stent therapy.

## 1. Introduction

Self-expanding esophageal stents (SEMS) are widely used in the management of malignant dysphagia, benign strictures, postoperative leaks, and spontaneous perforations. Their adoption has expanded over the past decades due to high technical success rates, rapid symptom relief, and the ability to restore oral intake in complex esophageal pathology, particularly in palliative oncology care [1]. Despite these advantages, SEMS are associated with a range of complications, among which stent migration remains the most frequent, reported in 20–40% of cases depending on stent design and underlying disease [2,3].

Migration is traditionally viewed as a largely mechanical issue—an undesirable but often non-emergent event that may be managed electively by retrieval or repositioning. As a result, most published work focuses on migration rates, risk factors, or technical preventive strategies such as stent fixation [4,5,6,7]. However, emerging evidence shows that late migration—occurring weeks to years after stent placement—can precipitate severe, life-threatening emergencies, far beyond simple recurrent dysphagia. These presentations are poorly characterized in existing literature, scattered across isolated case reports and lacking systematic synthesis.

Emergencies following late migration include small bowel obstruction, gastrointestinal perforation, peritonitis, and hemorrhagic events such as aorto-esophageal fistula, a condition with a mortality rate approaching 100% [8]. Thoracic and mediastinal complications are equally dramatic, with reported cases of esophageal-pleural fistula, tension pneumothorax, esophago-pericardial fistula, and sepsis. Airway-related emergencies represent another uniquely dangerous subtype. Proximal migration or erosion may result in tracheal obstruction, broncho-esophageal fistula, or even complete loss of airway, events that require immediate intervention and have been described almost exclusively in high-risk, often irradiated patients [9].

Although these presentations are rare, our aggregated case-level dataset highlights that they represent a clinically meaningful and under-recognized subset of stent-related morbidity [10,11,12]. Importantly, many of these events occur months to years after placement, frequently in patients considered “stable” or no longer under close endoscopic surveillance. Risk factors repeatedly identified across reports include prior radiation therapy, fully covered stent design, placement across the gastroesophageal junction, tumor regression, long indwelling times, and altered post-surgical anatomy such as after sleeve gastrectomy or gastric conduit reconstruction [5].

Despite the severity of these complications, no systematic review to date has specifically examined acute medical and surgical emergencies precipitated by late esophageal stent migration. Existing reviews discuss migration generically, with minimal attention to its potential to cause airway collapse, peritonitis, hemorrhage, or multiorgan sepsis. Consequently, clinicians may underestimate the urgency of new abdominal, respiratory, or thoracic symptoms in patients with prior esophageal stenting. The aim of this systematic review is to present the full spectrum of acute clinical presentations resulting from late esophageal stent migration, including intestinal obstruction, gastrointestinal perforation, thoracic and mediastinal fistulas, airway compromise, sepsis, and vascular erosion. By synthesizing all reported cases across published literature, this study seeks to define common patterns, mechanisms, risk factors, diagnostic features, and patient outcomes in order to improve clinical awareness, inform surveillance protocols, and guide rapid identification and management of these rare but potentially fatal complications.

## 2. Materials and Methods

### 2.1. Protocol and Registration

This systematic review was conducted in accordance with the Preferred Reporting Items for Systematic Reviews and Meta-Analyses statement [13] (Supplementary Table S1). The review protocol was prospectively registered in the PROSPERO international prospective register of systematic reviews (Registration ID: CRD420251123685).

### 2.2. Eligibility Criteria

Two investigators independently searched PubMed/MEDLINE, Embase, and Scopus from 1 January 1990 to 4 May 2026. The PubMed search string was as follows: ((“Esophageal Stents”[MeSH] OR “esophageal stent”[tiab] OR “self-expanding esophageal stent”[tiab] OR SEMS[tiab]) AND (“Stents, Self-Expanding”[MeSH] OR stent migration[tiab] OR stent dislocation[tiab] OR stent displacement[tiab] OR migrated stent[tiab]) AND (complication[tiab] OR complications[tiab] OR adverse event*[tiab] OR obstruction[tiab] OR perforation[tiab] OR fistula[tiab] OR hemorrhage[tiab] OR bleeding[tiab] OR airway[tiab] OR respiratory[tiab] OR pneumothorax[tiab] OR peritonitis[tiab] OR sepsis[tiab] OR abscess[tiab])). The Embase search string was: (‘esophageal stent’/exp OR ‘esophageal stent’:ti,ab OR ‘self-expanding esophageal stent’:ti,ab OR ‘SEMS’:ti,ab) AND (‘stent migration’:ti,ab OR ‘stent dislocation’:ti,ab OR ‘stent displacement’:ti,ab OR ‘migrated stent’:ti,ab) AND (‘complication’:ti,ab OR ‘obstruction’:ti,ab OR ‘perforation’:ti,ab OR ‘fistula’:ti,ab OR ‘hemorrhage’:ti,ab OR ‘bleeding’:ti,ab OR ‘airway’:ti,ab OR ‘respiratory’:ti,ab OR ‘pneumothorax’:ti,ab OR ‘peritonitis’:ti,ab OR ‘sepsis’:ti,ab OR ‘abscess’:ti,ab). The Scopus search string was: TITLE-ABS-KEY ((“esophageal stent” OR “self-expanding esophageal stent” OR “SEMS”) AND (“stent migration” OR “stent dislocation” OR “stent displacement” OR “migrated stent”) AND (“complication” OR “obstruction” OR “perforation” OR “fistula” OR “hemorrhage” OR “bleeding” OR “airway” OR “respiratory” OR “pneumothorax” OR “peritonitis” OR “sepsis” OR “abscess”)). The search was restricted to publications in the English language. No restrictions were applied regarding study design; case reports, case series, letters, and conference abstracts were all considered for inclusion, provided they met the eligibility criteria. Unpublished literature and grey literature were not searched. Any controversy was resolved by the intervention of a senior investigator. Reference lists of all included studies were manually screened to identify additional potentially eligible reports not captured by the electronic database searches. No formal forward citation tracking was performed. Where data on specific variables were unavailable or incompletely reported, the corresponding fields were recorded as missing and excluded from the denominator when calculating proportions.

#### Eligibility Criteria—Inclusion and Exclusion

Inclusion criteria:

- Case reports, case series, or observational studies describing at least one patient with an esophageal stent (metallic or plastic) placed for any indication.
- Documented evidence of stent migration or displacement occurring at least 4 weeks after initial placement.
- Presentation with an acute complication: e.g., intestinal obstruction, gastrointestinal or thoracic perforation, hemorrhage (massive bleeding), fistula formation (esophag-opleural, esophago-pericardial, tracheo-esophageal, aorto-esophageal), airway compromise, sepsis, pneumothorax, empyema, or other life-threatening event.
- Sufficient clinical, radiological, endoscopic or surgical detail to confirm both migration and the resulting complication.

Exclusion criteria:

- Reports with migration discovered on routine follow-up but without any acute or high-acuity clinical presentation (e.g., asymptomatic, mild dysphagia only).
- Early migration (< 4 weeks after placement)
- Reviews without primary patient-level data; non-original data.

For each included case report or series, data was extracted independently into a pre-designed Microsoft Excel spreadsheet. The extracted variables comprised: demographic information (age, sex); indication for stent placement (malignant vs. benign disease, type of pathology—stricture, leak/perforation, or other); stent characteristics (type—metallic SEMS or plastic, coverage status: fully covered vs. partially/uncovered, manufacturer, length and diameter, fixation method if applied, and anatomical location of placement—proximal, mid, distal esophagus or across the gastro-esophageal junction). We also recorded the interval from stent placement to migration or complication (in weeks or months), the type of migration (distal vs. proximal; intact stent vs. fractured), and the nature of the acute complication (intestinal obstruction, perforation, fistula, hemorrhage, airway compromise, or other). Additional extracted data included the diagnostic modality used (e.g., CT, X-ray, endoscopy, ultrasound), the management strategy (endoscopic retrieval, surgery, vascular repair, supportive therapy), and patient outcome (survival, morbidity, death, follow-up).

Numerical variables are presented as mean, SD or median with range, whereas categorical ones as frequency and valid percentages. Several studies lacked data on all variables of interest, and therefore, rates were estimated based on available data. Statistical analysis was carried out using IBM SPSS Statistics for Windows, Version 28.0 (IBM Corp., Armonk, NY, USA).

### 2.3. Risk of Bias Assessment

The methodological quality of included studies was assessed according to study design. Case reports and case series were evaluated using the Joanna Briggs Institute (JBI) critical appraisal checklist. Quality assessments were performed independently by two reviewers, and disagreements were adjudicated by a third reviewer.

### 2.4. Data Synthesis

Given the heterogeneity of study designs and outcome definitions, a quantitative meta-analysis was not planned a priori. Results were synthesized narratively, with descriptive statistics (counts, proportions, means or medians with ranges) used to summarize the frequency and types of late complications, their timing, and associated outcomes.

## 3. Results

**Figure 1:**
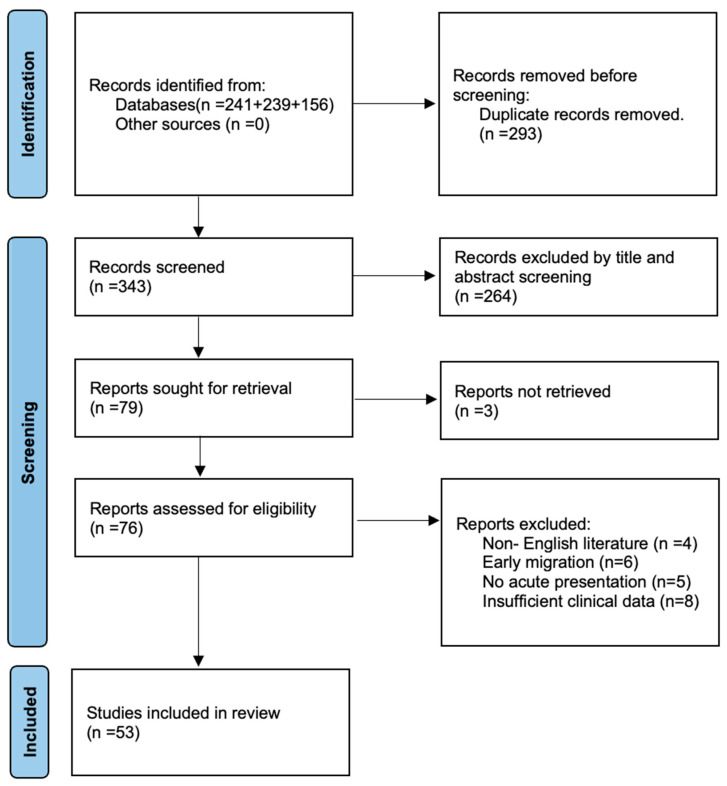
Trial flow of this systematic review.

The present systematic review includes 53 studies out of 343 unique articles, reporting on 53 patients with acute presentation after late esophageal stent migration [8,9,12,14,15,16,17,18,19,20,21,22,23,24,25,26,27,28,29,30,31,32,33,34,35,36,37,38,39,40,41,42,43,44,45,46,47,48,49,50,51,52,53,54,55,56,57,58,59,60,61,62,63]. The study selection process, including the number of records screened and the reasons for full-text exclusions, is summarized in Figure 1. The studies consist of 53 patients with a male: female ratio of 1.25:1. The mean age of the cohort was 59.8 ± 14.76 (mean, SD).

The indications for esophageal stent placement in the 53 analyzed cases were predominantly stenosis and dysphagia due to advanced esophageal cancer (32 cases, 60.3%). Benign conditions accounted for a smaller proportion, including 7 cases (13.2%) of GERD-related strictures, 3 cases (5.7%) of Boerhaave syndrome, and 2 cases (3.7%) of staple-line leak following sleeve gastrectomy. Postoperative strictures were also noted in 4 patients (7.4%). Additionally, 4 cases (7.4%) were associated with radiation-induced esophageal stenosis, and 1 case (1.9%) involved a benign stricture following caustic ingestion. An analytical overview of stent placement indications is presented in Table 1.

In the 53 included cases, all acute complications followed late migration, defined as events arising ≥4 weeks after esophageal stent placement, with a median time to presentation of 3 months and a range of 1–48 months. Among these, exact timing data were available for 48 cases. Stratifying these by latency interval, the majority (35 cases, 72.9%) presented between 1 and 6 months after stent placement. An additional 7 cases (14.6%) occurred between 6 and 12 months, while 6 cases (12.5%) presented more than one year after initial stent deployment, including some as late as 4 years. This distribution highlights that although most acute complications cluster within the first 6 months, a significant minority emerge after prolonged indwelling times.

Late esophageal stent migration exhibited a wide anatomical range (Table 2), with the gastrointestinal tract being the most commonly affected system, accounting for 64.2% of all reported cases (n = 34). This group encompassed a variety of small and large bowel sites, including the ileum, jejunum, duodenum, ileocecal valve, colon, rectum, and stomach, indicating a clear tendency for distally migrated stents to lodge in luminal digestive structures. The airway and respiratory tract comprised the second most frequent category, representing 15% of cases (n = 8), with migrations involving the trachea, bronchus, nasopharynx, hypopharynx, and proximal esophagus, all of which pose significant risks for respiratory compromise and airway emergencies. Thoracic complications were observed in 13.2% (n = 7) and included migration into the pleural cavity, chest wall, and pericardium, reflecting the potential for transmural erosion and mediastinal invasion. Vascular migration, though less common (5.7%, n = 3), involved critical sites such as the aortic arch and left common carotid artery and represented the most acutely life-threatening subset due to the risk of catastrophic hemorrhage. Lastly, rare solid organ involvement was seen in about 2% (n = 1), with one reported case of splenic migration.

The clinical manifestations of late esophageal stent migration correlated closely with the anatomical site of stent displacement. Stents that migrated into the small intestine—particularly the ileum and jejunum—were predominantly associated with small bowel obstruction (16 cases, 30.2%) and small bowel perforation (4 cases, 7.5%). These presentations reflect the narrow luminal diameter and prolonged transit time that predispose to impaction and transmural injury. Similarly, stents lodged in the colon and rectum accounted for cases of large bowel obstruction (3 cases, 5.7%), colonic perforation with peritonitis (1 case, 1.9%), rectal bleeding (1 case, 1.9%), and anal pain (2 cases, 3.8%), often with visible stent extrusion. Stent migrating into thoracic or airway structures, such as the trachea, bronchi, hypopharynx, and nasopharynx, were typically associated with respiratory compromise. These cases manifested as pneumonia, respiratory distress, or sepsis in 11 patients (20.8%), emergent airway obstruction in 1 patient (1.9%), and pneumothorax in another (1.9%). Migration into the pleural cavity or pericardium similarly produced thoracic complications, including chest pain and pericardial inflammation.

Gastric stent migration was linked with gastric perforation or peritonitis (2 cases, 3.8%) and severe epigastric pain (4 cases, 7.5%), underscoring the stomach’s vulnerability to both pressure necrosis and foreign body trauma. Vascular involvement, though rare, carried high risk: one patient developed a symptomatic pseudoaneurysm (1.9%) and another presented with hemodynamic instability (1.9%), both likely due to erosion into major vessels such as the aortic arch or carotid artery. Additionally, a case of gastrocutaneous fistula with sepsis (1.9%) reflected cutaneous externalization of a migrated stent, indicative of chronic transmural erosion. Collectively, these data confirm that the anatomical destination of a dislodged stent strongly predicts the nature and severity of clinical presentation. An overview of clinical presentations associated with late esophageal stent migration is presented in Table 3.

Diagnosis of acute complications following late esophageal stent migration was established using multiple diagnostic modalities, with computed tomography (CT) as the primary imaging method. CT scans were employed in the majority of cases (n = 37, 69.8%). Plain abdominal and chest radiographs were frequently used as initial tools, particularly when stent migration was suspected based on symptom onset or physical examination. Among the 53 cases, 39 underwent endoscopic assessment—most commonly upper gastrointestinal endoscopy—which provided direct visualization of the stent’s position, mucosal injury, or fistula orifice. Bronchoscopy was performed in 14 patients, especially in cases involving airway symptoms, and enabled detection of tracheobronchial invasion or stent erosion into the airway. In select cases, additional radiological modalities such as angiography, ultrasound (including point-of-care ultrasound), and barium swallow were used to complement diagnostic clarity.

Treatment strategies for acute complications due to late esophageal stent migration were diverse and largely dictated by anatomical site and severity of presentation. Surgical intervention was the most frequently employed approach, with enterotomy and stent retrieval followed by primary bowel repair performed in 19 cases (35.8%). Upper gastrointestinal endoscopy was employed in 11 cases (20.8%) for direct retrieval in accessible locations; this included 1 case managed via single-balloon enteroscopy and 2 cases via double-balloon enteroscopy to reach stents located in deeper segments of the small intestine. Manual stent extraction, typically through the anal canal was reported in 3 cases. More complex interventions included endovascular aortic repair (n = 2) in cases of vascular erosion and colonoscopic retrieval (n = 2) in colonic migration. A small number of patients (n = 2) were managed conservatively due to advanced malignancy or poor performance status, while 2 patients died before any intervention could be undertaken. Rare procedures included gastrotomy or colotomy with stent removal, hemicolectomy, tracheostomy or bronchoscopy for airway obstruction, and combinations of thoracotomy, esophagectomy, and reconstructive procedures for mediastinal or airway fistulas. An overview of intervention types employed is presented in Table 4.

Among the 53 patients, 15 deaths were recorded, corresponding to an overall mortality rate of 28.3%. Eight patients (15.1%) died shortly after presentation due to catastrophic events such as hemorrhage, respiratory failure, or fulminant sepsis. Two additional patients died within the first postoperative week, one from bronchopneumonia following laparotomy for small bowel obstruction and one from respiratory failure after enterotomy for ileocecal valve obstruction. Two patients experienced delayed mortality: one from rebleeding after aorto-esophageal fistula on postoperative day 26 despite TEVAR, and another from sepsis following laparotomy for small bowel perforation on postoperative day 55. One patient died later during follow-up due to progression of underlying malignancy, not directly related to the stent complication. One patient with pleural cavity migration and one with tracheoesophageal fistula died from disease progression during palliative follow-up.

Mortality was highest among patients with vascular and airway involvement. Both cases of aorto-esophageal fistula resulted in death despite intervention, reflecting the near-universally fatal nature of this complication. Tracheal involvement was associated with fatal outcomes in 4 of 5 patients, primarily due to airway obstruction, hemorrhage, or aspiration. Pleural complications were fatal in 2 of 3 patients. One patient with esophago-pericardial fistula and one with broncho-esophageal fistula died during the same hospitalization despite attempted endoscopic management. Among survivors, no additional major long-term morbidity directly attributable to the migrated stent was reported beyond the acute event and its immediate management.

Study quality was assessed using the Joanna Briggs Institute (JBI) critical appraisal checklist for case reports (Supplementary Table S2). Overall, the included studies demonstrated a low risk of bias, with a high mean JBI score of 7.88 ± 0.46, indicating generally robust and comprehensive reporting. The majority of case reports fulfilled all or nearly all appraisal criteria. Nevertheless, five reports failed to meet two checklist items, most commonly related to incomplete documentation of post-intervention clinical outcomes or follow-up.

## 4. Discussion

Self-expanding esophageal stents (SEMS) play a pivotal role in the management of both malignant and benign esophageal conditions. They are widely employed to palliate malignant dysphagia in advanced esophageal cancer and are also utilized in benign scenarios such as refractory strictures, esophageal perforations, and post-surgical leaks [61]. The endoscopic placement of SEMS provides rapid relief of obstructive symptoms and can effectively seal perforations or leaks, offering a minimally invasive therapeutic approach with prompt restoration of oral intake. Overall, SEMS placement has demonstrated a favorable safety profile, with high technical success rates and relatively infrequent serious complications—the most common being stent migration, typically manageable through endoscopic retrieval [62]. This balance of efficacy and safety has established SEMS as a cornerstone in the treatment of esophageal diseases [63].

However, while most migrations are asymptomatic or detected early, late migration—defined as stent displacement occurring 4 weeks or more after insertion—can result in a spectrum of acute, often life-threatening complications. Our systematic review identified numerous reports of stents migrating weeks to months after insertion and causing severe outcomes, including esophageal or gastrointestinal perforation, formation of fistulous tracts (e.g., tracheo-esophageal, broncho-esophageal, gastro-pleural, or even aorto-esophageal fistulas), acute obstructions, and major hemorrhage. While stent migration itself is common (reported in roughly 10–30% of cases), the majority of migrations are clinically silent [5]. However, a minority present acutely with serious complications that carry high morbidity and mortality. In earlier series of malignant stent cases, up to two-thirds of patients experienced some delayed complication, and about 16% died due to stent-related issues [64].

The mechanisms underlying these late migration complications are based on the interaction between the indwelling stent, the esophageal wall, and adjacent organs. Over time, even a well-positioned stent can exert pressure on the esophageal mucosa, leading to pressure necrosis and erosion into surrounding structures [65]. A migrating stent may act as a foreign object with its edges or wires directly traumatizing tissue as it moves. For example, a slowly migrating esophageal stent can eventually erode through the esophageal wall and create a tract into the trachea or bronchus, resulting in a tracheoesophageal fistula [65]. Such fistulas often develop weeks or months after stent placement due to gradual tissue ischemia at pressure points. Similarly, prolonged contact can lead to erosion into major blood vessels—the classic instance being aortic erosion and fistula, where the stent (or the tumor it is palliating) wears into the aortic wall, typically after weeks of indwelling time and often in the context of prior radiation therapy [65]. Radiation and chemotherapy are known to weaken tissue integrity and impair healing, so a stent in a previously irradiated esophagus is more prone to cause late perforation or fistulation of the friable tissue [65]. Anatomical changes after the initial stent placement also contribute to late migration. Tumor shrinkage (in malignant cases) or healing of a benign stricture can create a lumen wider than the stent, reducing friction and allowing the stent to dislodge [65,66]. In benign conditions, once inflammation subsides or a perforation seals, a fully covered stent may lose its purchase and be propelled distally by esophageal peristalsis [67]. Surgical alterations can further predispose to migration; for instance, resection of the gastroesophageal junction or pylorus will remove the natural distal barrier, facilitating the stent’s migration into the stomach or small intestine [12]. In summary, late complications typically arise from a combination of mechanical forces and biological vulnerability: the constant radial force and peristaltic movement of the stent can lead to erosion (especially if tissues are weakened by radiation, chemotherapy, or tumor necrosis), and any decrease in tumor bulk or postoperative change in anatomy can precipitate stent dislocation into areas where it was not intended to be. These insights explain why late stent migrations can breach adjacent structures, resulting in fistulas to the airway or great vessels, or travel distally to cause obstruction or perforation in the GI tract.

It is worth noting that benign vs. malignant indication influences the nature of complications observed: stents for benign conditions (e.g., benign strictures, postsurgical leaks) are typically left in for a limited duration, but they are often fully covered and thus at high risk of migrating; the complications in benign cases tend to be related to the migration itself—for instance, a stent causing intestinal obstruction or perforation after moving distally [12]. In malignant cases, stents may remain indefinitely, so late complications like tissue erosion (fistula, hemorrhage) are more common, especially if the tumor progresses or the patient has received radiation [65]. In summary, risk factors for late migration include use of fully covered or plastic stents, smaller stent diameter, location across the GE junction or in a surgically altered anatomy, and an initially large tumor burden that later regresses. Risk factors for severe outcomes include prior radiation therapy, certain stent designs (lacking anti-migration features or exuding high radial force), and proximal tumor location [66]. Awareness of these factors can help in stent selection and in identifying high-risk patients who warrant closer follow-up. It should be noted that these observations are descriptive in nature and derived from aggregated published case reports. They do not constitute formal risk-factor analysis, and the frequencies cited should not be interpreted as true incidence estimates or used to establish causal relationships.

Recognizing and diagnosing acute complications of late stent migration can be challenging, in part because symptoms often present insidiously or mimic other conditions. As noted, many stent migrations are initially asymptomatic—the stent may move from its original position without immediate obvious effect, especially if it migrates into the stomach. Patients might have only vague symptoms or no symptoms until a complication (like a perforation or obstruction) reaches an advanced stage. Moreover, the patency of a hollow stent can allow some passage of food or secretions even after migration, potentially masking an evolving problem. This phenomenon—the stent’s lumen maintaining some flow despite misplacement—means clinicians must have a high index of suspicion rather than relying on classic obstruction signs.

When patients with esophageal stents do develop new symptoms, these can be nonspecific. Chest pain, odynophagia, worsening dysphagia, unexplained fever or sepsis, hematemesis, or respiratory symptoms (cough, dyspnea) should all raise concern about a possible stent complication. Importantly, seemingly unrelated complaints can be clues: there are cases of patients presenting with aspiration pneumonia or respiratory distress that were eventually found to have a tracheo-esophageal fistula from a migrated esophageal stent. Notably, any respiratory distress in a patient with an esophageal stent should prompt an evaluation for stent migration or a fistulous connection between esophagus and airway [59]. Similarly, a patient with a stent who develops gastrointestinal bleeding—even a small “sentinel” bleed—may be showing the first sign of an impending major hemorrhage from an erosion into a blood vessel. This pattern (sometimes called Chiari’s triad in aorto-esophageal fistula) involves a minor bleed or sentinel hematemesis followed by a quiescent interval, and then catastrophic hemorrhage. Thus, even minor bleeding in a stented patient must be taken seriously and investigated promptly.

Optimal imaging is critical for diagnosis. A plain chest or abdominal radiograph can often confirm that a stent has migrated from its original location. In our review, simple X-ray was frequently the first clue to migration. However, cross-sectional imaging is usually required to fully delineate the complication. Computed tomography with contrast is the modality of choice when a perforation, fistula, or abscess is suspected, as it can show extraluminal air, fluid collections, and the relationship of the stent to adjacent structures. CT angiography is especially valuable if a vascular involvement (e.g., an aortic fistula) is in question. Endoscopy can also play a diagnostic role—for example, if a patient’s stent is not seen in place on endoscopy, one must assume it has migrated distally; conversely, endoscopy might directly visualize a fistula or ulcer caused by the stent. Caution is warranted during endoscopy in cases of suspected fistula to a large vessel, as insufflation or instrument manipulation could precipitate bleeding.

Management of acute complications from late stent migration must be individualized, considering the nature of the complication, the patient’s condition, and the underlying esophageal disease. Broadly, interventions can be divided into endoscopic approaches, surgical (or other invasive) approaches, and combinations thereof.

Endoscopic retrieval or repositioning is often the first-line approach for a migrated stent. If a stent migrates without causing significant perforation, it can frequently be removed endoscopically with graspers or snares. In fact, migrated esophageal stents located in the stomach or intestine should be removed endoscopically whenever possible [7]. Case series have reported high success rates for endoscopic stent retrieval—one review noted that all migrated stents in their cohort were successfully removed endoscopically with no resulting injury [68,69]. Advanced techniques such as overtube-assisted retrieval, double-balloon enteroscopy (for stents that travel deep into the small bowel), or fluoroscopic guidance may be employed [69,70]. In many instances, this can be performed emergently at bedside or endoscopy suite, and it immediately eliminates the foreign body. If the migration is partial (e.g., the stent has shifted but not completely dislodged), an endoscopist may sometimes reposition the stent or deploy a new stent to bridge a developing complication [7]. For example, a partially migrated stent that leaves a recurrent esophageal tear might be overlapped with a second stent to re-cover the lesion [7]. It should be noted, however, that if a stent has fractured or become embedded, endoscopic removal can be challenging and may require fragmentation of the stent or adjunct tools. Nonetheless, the endoscopic route avoids open surgery and is preferred whenever feasible, especially in patients with significant comorbidities.

Despite best efforts, many complications demand surgical intervention or other invasive procedures. Stent migrations that result in perforation of the esophagus or bowel often require emergency surgery. Free perforation into the mediastinum or pleural cavity mandates surgical repair or drainage to source-control the contamination (such as an esophagectomy or primary repair with drainage of an abscess/empyema). In our review, when a stent migrated through the esophageal wall into the pleural space, the patient underwent a thoracotomy to extract the stent and repair the defect [35]. Similarly, stent-induced perforations of the stomach or small intestine usually require laparotomy; the migrated stent and any non-viable bowel tissue must be removed. This highlights that by the time a migration leads to perforation and peritonitis, the physiological insult is severe. Prompt surgery is life-saving, but outcomes hinge on how early the problem is recognized and the patient’s baseline reserves.

In the case of tracheo-esophageal or broncho-esophageal fistulas, management can be especially complex. If the patient has a reasonable life expectancy (e.g., a benign TEF or a potentially curable cancer), surgical repair of the fistula with muscle flap reinforcement is often the definitive solution [27]. However, many TEFs associated with esophageal stents occur in the setting of advanced malignancy or poor surgical candidacy. In those situations, a dual stenting approach is commonly used for palliation: an esophageal covered stent to seal off the esophagus, and in some cases, a tracheobronchial stent to stabilize the airway. Covered esophageal stents have been shown to effectively close malignant TEFs in a high percentage of patients, providing relief of aspiration and allowing enteral nutrition [71]. Our review reflects this practice –reports documented successful palliation of TEF by placing a new esophageal stent across the fistula, even when a prior stent had initially caused the complication. In one series of 170 esophageal stent cases, the authors noted that stenting nearly completely relieved tracheoesophageal fistulas and significantly improved quality of life in those patients [72]. Airway stenting (with a silicone or metallic Y-stent, for example) is added if needed to support the airway and prevent airway collapse or stent intrusion. Endoscopic vacuum therapy has also emerged as a possible alternative for some esophageal leaks/fistulas, but data in the context of stent-induced fistulas are still limited. Ultimately, a tailored combination of therapies is often required. An example from our review involved a patient who needed an urgent tracheostomy in the presence of an unrecognized stent-induced TEF: the solution entailed using a bronchoscope and balloon to dilate the tracheal lumen inside the esophageal stent and safely place a tracheostomy tube, since conventional methods were impossible with the stent protruding into the airway [59]. This case underscores the ingenuity sometimes required to manage stent complications—involving thoracic surgeons, gastroenterologists, and interventional pulmonologists in a multidisciplinary approach.

Perhaps the most feared complication is an aorto-esophageal fistula or major vessel erosion leading to hemorrhage. This situation is a surgical emergency and often fatal. Management typically demands a dual approach: controlling the bleeding vessel and repairing the esophageal defect. Thoracic aortic erosion (primary AEF) is now frequently managed with thoracic endovascular aortic repair (TEVAR)—essentially placing a covered vascular stent graft in the aorta to seal the fistula from the vascular side. This can temporarily or definitively stop the hemorrhage. Concurrently, the esophagus may be stented (to tamponade bleeding and prevent stent contamination) or surgically addressed (esophageal diversion or resection), depending on the scenario. Despite aggressive management, outcomes remain poor. Aorto-esophageal fistula carries an almost 100% mortality if untreated, and even with state-of-the-art intervention, reported mortality rates remain as high as 70–80% [73]. Notably, both patients with aorto-esophageal fistula in our cohort succumbed to the condition despite prompt recognition and multidisciplinary management. Our review did note a few cases of successful salvage—for instance, one patient with a stent-induced carotid artery pseudoaneurysm (a form of vascular erosion) survived after endovascular coil embolization of the carotid and surgical removal of the stent. Such cases are rare, and rapid recognition is the key: a sentinel bleed in a stented patient should prompt CT angiography and urgent involvement of a multidisciplinary team including cardiovascular surgeons and interventional radiologists. In practice, if an AEF is suspected, one might immediately deploy a covered esophageal stent to tamponade bleeding (if available) while preparing for definitive aortic intervention—essentially a damage control approach [74]. Randomized trials are not feasible in this clinical scenario; however, accumulated case experience suggests that combined esophageal and aortic stenting can sometimes achieve short-term survival, allowing later surgical repair if the patient is stable [75]. Nonetheless, the prognosis is grave: even in-hospital, such fistula often proves fatal within hours.

In summary, the treatment of late stent migration complications ranges from relatively simple endoscopic procedures (for contained migrations without tissue damage) to highly complex surgical rescues. Outcomes are variable. When diagnosed early and managed endoscopically, patients often do well and avoid major morbidity [61]. On the other hand, once a serious complication like a perforation, fistula, or hemorrhage has occurred, the mortality rate is significant. Our findings align with prior observations that stent-related esophago-respiratory fistulas carry a poor prognosis (case fatality reported around 20–50% even with intervention) and aorto-enteric fistulas even higher [73,76]. Multidisciplinary care is crucial: these patients may require critical care support, interventional radiology (for angiographic embolization or stent-grafts), cardiothoracic surgery, and gastroenterology. Preventing these complications in the first place, or at least detecting them early, is therefore crucial. A concise overview of complication types, their likely mechanisms, preferred diagnostic modalities, and typical management strategies is provided in Table 5.

Given the potential for delayed catastrophic events, surveillance after esophageal stent placement should be regarded as an integral component of care rather than an optional adjunct. However, optimal surveillance strategies differ substantially between benign and malignant indications and must be individualized according to patient risk, life expectancy, and treatment intent.

In benign conditions, esophageal stents are typically placed as a temporary measure to facilitate healing of perforations, leaks, or refractory strictures. In this setting, prolonged indwelling time is a major determinant of late migration and erosion. Accordingly, most experts advocate planned stent removal once the therapeutic objective has been achieved, generally within 2–4 weeks, with earlier removal favored when feasible [77]. Structured follow-up during this interval -combining clinical assessment and confirmatory imaging or endoscopy— allows early detection of migration or mucosal injury and reduces the risk of delayed complications.

In malignant disease, stents are usually left in situ indefinitely for palliation, necessitating a different surveillance paradigm. Because routine endoscopic surveillance is often impractical and may impose unnecessary burden in patients with advanced cancer, follow-up relies primarily on vigilant clinical monitoring. Patients and caregivers should be educated to promptly report new or worsening symptoms, including chest pain, recurrent dysphagia, fever, hematemesis, cough, or respiratory distress, which may herald stent migration, erosion, or fistula formation.

Although no consensus exists regarding formal surveillance schedules in malignant cases, periodic imaging is commonly employed in clinical practice to confirm stent position and identify early complications. Interval chest radiography or fluoroscopic swallow studies—often performed within the first few weeks after placement and subsequently at longer intervals in asymptomatic patients —represent pragmatic, low-threshold tools for surveillance. The utility of routine imaging must be balanced against cost, radiation exposure, and the clinical context of oncologic patients, many of whom have limited functional reserve and short life expectancy [78,79]. Consequently, surveillance strategies should be proportionate and risk-adapted, prioritizing symptom-driven evaluation and minimally invasive monitoring.

Based on collective experience and the findings of this review, we recommend that all patients undergoing esophageal stenting have scheduled clinical follow-up and at least one imaging confirmation of stent position within the first weeks after placement. Additional imaging or endoscopic evaluation should be undertaken promptly if migration is suspected or symptoms arise. While frequent routine endoscopy is rarely justified, selective endoscopic assessment in the setting of concerning symptoms or equivocal imaging findings can be lifesaving. Patients with recognized risk factors —including prior chemoradiotherapy, proximal stent placement near the airway, fully covered stent design, or initiation of new oncologic therapies after stenting— may warrant closer surveillance. Tumor regression or treatment-related mucosal injury in these patients can compromise stent fixation and predispose to late migration, underscoring the importance of heightened clinical vigilance.

Our review highlights several important gaps for future research and innovation. First, there is a clear need for improved stent technology and design to address the persistent issue of migration. Despite various anti-migration features available (barbs, flared ends, anti-reflux valves, external fixation via endoclips, etc.), current stents still migrate at unacceptably high rates in certain contexts. No solution to date has been able to completely safeguard against migration or its complications. Future device development should focus on enhancing stent anchorage without increasing tissue injury—a challenging balance. Potential innovations include biodegradable stents, which naturally degrade after a predefined period and may reduce late erosion when long-term support is unnecessary, as well as patient-specific three-dimensional (3D)–printed stents, which may allow tailored geometry and radial force profiles adapted to individual esophageal anatomy. While clinical data remains limited, such customization has the potential to reduce migration and pressure-related injury associated with conventional off-the-shelf stents [80,81]. Additionally, novel coatings or materials might reduce pressure injury to the esophageal wall (for example, a softer polymer at the ends of a metal stent could reduce the risk of ulceration) [82]. Collaborative efforts between bioengineers, industry, and clinicians will be crucial to designing stents that stay in place securely but are also gentle on the esophagus.

Second, more research is needed to optimize clinical protocols: in particular, the ideal timing of stent removal in benign conditions, and the best practices for surveillance in malignancy. As discussed, practices vary regarding how long to leave a stent in a healed leak or stricture. Prospective studies or even randomized trials could compare, for example, removing a stent at 2 weeks versus 6 weeks for benign leaks to see differences in success and complication rates. Currently, recommendations (such as removal at 4 weeks for perforations) are based on retrospective data and expert opinion. Formal study could strengthen these guidelines. Likewise, establishing standardized follow-up schedules (imaging or endoscopy at set intervals) could be studied in a comparative effectiveness manner to determine if they reduce late complication incidence. A related research need is identifying biomarkers or early indicators of impending complications—for instance, is there a way to detect micro-perforation or early fistula formation (perhaps via CT imaging signs or endoscopic appearance) before it becomes overt. Answering these questions would likely require large multicenter registries, given the relatively low frequency of events like aorto-esophageal fistula.

Third, our findings call for better risk stratification tools. Future research could develop a scoring system or risk model for patients receiving esophageal stents, incorporating factors like radiation history, stent type, location, and patient comorbidities, to predict who is at highest risk for migration or severe complications. This would help tailor follow-up intensity and perhaps influence initial stent choice. For example, a patient with a high-risk profile might be a candidate for an alternative therapy (such as endoscopic vacuum therapy for a leak instead of a stent) to avoid the risk altogether, but robust data are needed to guide such decisions. Comparative studies of stents versus other modalities for managing benign esophageal injuries (leaks, perforations) would be valuable—if a non-stent approach yields fewer late complications, it might be preferable in certain cases.

Another area of research is the management of stent complications itself. Given the rarity of events like AEF or tracheobronchial fistula in the context of stents, most published experience is in single-case reports. There is an opportunity for pooled analyses or systematic reviews focusing specifically on these worst-case scenarios to distill which interventions have the best outcomes. For instance, a systematic review of all published aorto-esophageal fistula cases (regardless of cause) could better inform whether dual stenting (aortic and esophageal) offers a survival advantage over surgery, etc. As more cases are reported and technology (like improved endovascular stent-grafts) advances, outcomes might improve from the dismal figures of the past—but only if we systematically analyze and learn from each case.

Finally, prospective trials in the field of esophageal stenting should be encouraged. Many of the practices today are based on level II/III evidence. Randomized controlled trials (RCTs) could be feasible for questions like anti-migration techniques (e.g., stent + clipping vs. stent alone), or different stent designs in similar patient populations. It is promising that research interest is growing, while RCTs in this area can be challenging (given heterogeneous patient conditions), even well-designed observational studies and registries can provide high-quality evidence.

This systematic review is subject to several important limitations that must be acknowledged when interpreting its findings. First, the available evidence is derived exclusively from case reports, which inherently limits the strength of inference. Such publications are prone to reporting bias, as dramatic or unusual presentations—particularly fatal thoracic, airway, or vascular events—are more likely to be reported than uncomplicated or clinically silent migrations. Study quality was assessed using the JBI critical appraisal checklist for case reports (Supplementary Table S2), yielding a high mean score of 7.88 ± 0.46. It must be emphasized, however, that JBI compliance reflects the quality of reporting rather than the strength of evidence. Since virtually all included studies are case reports, the overall evidentiary strength remains low by definition, and the frequency distribution of complication types observed in this review should not be interpreted as reflecting true incidence rates. Second, there was heterogeneity in reporting quality, including inconsistent documentation of stent characteristics, timing of migration, diagnostic modalities, and therapeutic interventions. Key variables such as prior oncologic therapy, anatomical alterations, tissue condition, and the exact timing of symptom onset were inconsistently or incompletely reported in certain cases. In several reports, descriptors such as “weeks,” “months,” or “years later” lacked precise temporal quantification, limiting the ability to perform standardized temporal analysis beyond categorical grouping. This heterogeneity also precluded meaningful synthesis of associations between risk factors and specific complication types. Third, although risk factors such as prior radiotherapy, fully covered stent design, tumor regression, and altered surgical anatomy are frequently referenced across included reports, it must be emphasized that the review design does not permit causal inference. No true risk-factor analysis can be performed on the basis of aggregated case reports, and the frequencies observed among published cases must not be interpreted as incidence estimates in the stented population at large. The evidence presented is exclusively descriptive, and the associations discussed reflect patterns observed in a highly selected, publication-biased sample rather than verified epidemiological relationships. Fourth, the absence of standardized definitions for late migration and acute complication categories across studies further complicates comparison. Several reports used differing thresholds for defining late versus early migration, and some did not specify whether the complication was directly attributable to migration or to other concurrent esophageal pathology. Additionally, variation in imaging protocols and endoscopic practices across institutions limited the ability to compare diagnostic performance or modality-specific detection patterns. Furthermore, therapeutic decision-making was highly context-dependent and influenced by institutional expertise, local resources, and patient comorbidities. As a result, intervention success rates and survival outcomes reported here may be confounded by variables such as hemodynamic status, sepsis severity, performance status, or access to advanced airway or thoracic surgery services. The lack of denominator data —meaning the absence of total numbers of stented patients at risk— prevents calculation of incidence rates or risk estimates and restricts the conclusions to descriptive associations. Finally, the retrospective nature of the included cases and the reliance on narrative descriptions introduce the potential for selective data omission, incomplete follow-up, and inconsistency in outcome reporting. Mortality attribution, in particular, was not always clearly delineated as being directly due to the migrated stent versus underlying malignancy or postoperative complications. These factors limit the generalizability of the findings and underscore the need for prospective multicenter registries or standardized reporting frameworks to more accurately characterize the natural history, risk factors, and optimal management strategies for late stent migration.

In conclusion, while late esophageal stent migration and its complications are relatively infrequent, their potential for substantial morbidity and mortality warrants heightened clinical vigilance. This systematic review synthesizes available evidence on these catastrophic events, highlighting diagnostic challenges and significant gaps in long-term surveillance. Advancements in stent design, implementation of risk-adapted and structured post-placement follow-up strategies, improved patient stratification, and systematic evaluation of outcomes may collectively mitigate the incidence and severity of late stent-related complications. While the overall benefit–risk balance of esophageal stenting remains strongly favorable, given its established efficacy in palliation of dysphagia and management of leaks, ongoing refinement of clinical practice by surgical and gastroenterological communities is essential to optimize long-term patient safety and prevent fatal outcomes, particularly in high-risk populations.

## 5. Conclusions

Late migration of esophageal stents, although uncommon, can result in acute, life-threatening complications with substantial morbidity and mortality, often occurring months to years after placement in patients no longer under close surveillance. This systematic review suggests that early recognition, prompt imaging, and multidisciplinary management remain clinically important for improving outcomes, particularly when airway or vascular structures are involved. Risk-adapted surveillance strategies, along with continued advances in stent design and prospective research, are warranted to further enhance the long-term safety of esophageal stent therapy, though formal recommendations await validation through prospective studies.

## Figures and Tables

**Table 1:** Indications for stent placement in our systematic review.

Indication for Stent Placement	Number of Cases	Percentage
Malignant stenosis/dysphagia	32	60.3%
Esophageal adenocarcinoma	17	32.1%
Squamous cell esophageal cancer	14	26.4%
Not specified esophageal cancer	1	1.9%
Benign strictures/stenosis (GERD-related)	7	13.2%
Postoperative strictures	4	7.5%
Post- esophagectomy	3	5.7%
Post-esophageal perforation repair	1	1.9%
Radiation-induced strictures	4	7.5%
Lung cancer	2	3.8%
Laryngeal cancer	1	1.9%
Tongue cancer	1	1.9%
Boerhaave syndrome	3	5.7%
Staple-line leak after sleeve gastrectomy	2	3.8%
Caustic injury-related stricture	1	1.9%

**Table 2:** Anatomical distribution of late esophageal stent migration sites in our systematic review.

Anatomical Group	Migration Site	Number of Cases	Percentage
Airway/Respiratory Tract	Main Trachea	5	9.4%
	Right Bronchus	1	1.9%
	Nasopharynx	1	1.9%
	Hypopharynx	1	1.9%
Gastrointestinal Tract	Proximal Esophagus	1	1.9%
	Stomach	6	11.3%
	Duodenum	1	1.9%
	Jejunum	5	9.4%
	Ileum	11	20.8%
	Ileocecal Valve	3	5.7%
	Colon	3	5.7%
	Rectum	3	5.7%
	Anal Canal	1	1.9%
Thoracic Compartment	Chest Wall	2	3.8%
	Pericardium	2	3.8%
	Pleural Cavity	3	5.7%
Vascular Structures	Aorta	2	3.8%
	Left Common Carotid Artery	1	1.9%
Solid Organs	Spleen	1	1.9%

Note: Each patient was assigned to a single anatomical migration site corresponding to the primary location of stent displacement reported in the literature.

**Table 3:** Clinical Presentations Associated with Late Esophageal Stent Migration.

Affected System	Clinical Presentation	Number of Cases	Percentage
Gastrointestinal	Small bowel obstruction	16	30.2%
	Small bowel perforation	4	7.5%
	Epigastric pain	4	7.5%
	Large bowel obstruction	3	5.7%
	Gastric perforation/peritonitis	2	3.8%
	Anal pain	2	3.8%
	Gastrocutaneous fistula/sepsis	1	1.9%
	Rectal bleeding	1	1.9%
	Colonic perforation/peritonitis	1	1.9%
Respiratory/Thoracic	Pneumonia/respiratory distress/sepsis	11	20.8%
	Acute pneumothorax	1	1.9%
	Emergent airway obstruction	1	1.9%
	Chest pain	1	1.9%
Vascular/Hemodynamic	Hematemesis	3	5.7%
	Symptomatic pseudoaneurysm	1	1.9%
	Hemodynamic instability	1	1.9%

Note: Each patient contributed a single clinical presentation, corresponding to the primary or most acute manifestation reported in the literature.

**Table 4:** Management strategies for acute complications related to late esophageal stent migration.

Intervention Type	Number of Cases	Percentage
Enterotomy with stent retrieval and primary repair	19	35.8%
Upper GI endoscopy	11	20.8%
Manual extraction	3	5.7%
Endovascular aortic repair	2	3.8%
Colonoscopy retrieval	2	3.8%
Died before any intervention	2	3.8%
Conservative management due to advanced malignancy/age	2	3.8%
Gastrotomy with stent retrieval and primary repair	1	1.9%
Right hemicolectomy	1	1.9%
Colotomy with stent retrieval and primary repair	1	1.9%
Bilateral myringotomy with stent retrieval and grommet insertion	1	1.9%
Esophagostomy and jejunostomy	1	1.9%
Transarterial chemoembolization (TACE) of the 5th intercostal artery	1	1.9%
Bronchoscopy and tracheostomy	1	1.9%
Laryngoscopy with stent retrieval	1	1.9%
Cervical esophagostomy	1	1.9%
Thoracotomy with esophagectomy and tracheobronchial defect repair (serratus flap)	1	1.9%
Embolism control, splenectomy, and Roux-en-Y gastrojejunostomy	1	1.9%
Thoracotomy, pericardial window, gastrostomy, and tracheostomy	1	1.9%

**Table 5:** Summary of complication types, underlying mechanisms, preferred diagnostic modalities, and typical management strategies in late esophageal stent migration.

Complication Type	Likely Mechanism	Preferred Diagnostic Modality	Typical Management
Small bowel obstruction	Distal migration with luminal impaction at narrow bowel segments	CT abdomen; plain abdominal radiograph	Enterotomy with stent retrieval; double-balloon enteroscopy for accessible cases
Gastrointestinal perforation	Pressure necrosis and transmural erosion by stent edges	CT abdomen with contrast	Emergency laparotomy; bowel resection if non-viable tissue
Gastric perforation/ peritonitis	Direct mucosal trauma and pressure necrosis at gastric wall	CT abdomen with contrast	Laparotomy; gastrotomy with stent retrieval and primary repair
Tracheoesophageal/ broncho-esophageal fistula	Gradual erosion through esophageal wall into airway; tissue ischemia at pressure points	CT chest; bronchoscopy	Dual stenting for palliation; surgical fistula repair with muscle flap in operable patients
Airway obstruction	Proximal migration with mechanical occlusion of trachea or bronchus	Bronchoscopy; CT chest	Emergency bronchoscopy; tracheostomy; laryngoscopy with stent retrieval
Esophago-pleural fistula	Transmural erosion through esophageal wall into pleural space	CT chest with contrast	Mini-thoracotomy; stent retrieval with pleural drainage
Esophago-pericardial fistula	Transmural erosion into pericardium; mediastinal invasion	CT chest with contrast; echocardiography	Thoracotomy with pericardial window; multidisciplinary surgical approach
Aorto-esophageal fistula	Chronic pressure erosion into aortic wall; potentiated by prior radiotherapy	CT angiography	TEVAR for vascular control; covered esophageal stent as bridge; combined intervention
Carotid artery pseudoaneurysm	Stent erosion into carotid artery wall	CT angiography	Endovascular coil embolization; surgical stent removal
Splenic migration	Extraluminal migration via transmural erosion into solid organ	CT abdomen with contrast	Splenectomy; Roux-en-Y reconstruction

## Data Availability

The raw data supporting the conclusions of this article will be made available by the authors on request.

## Supplementary Materials

The following supporting information can be downloaded at: https://www.mdpi.com/article/10.3390/jcm15135092/s1, Supplementary Table S1: PRISMA Checklist; Supplementary Table S2: JBI Quality Assessment Table.

## Abbreviations

The following abbreviations are used in this manuscript:

AEF	Aorto-esophageal fistula
CT	Computed tomography
ESGE	European Society of Gastrointestinal Endoscopy
GE	Gastroesophageal
GERD	Gastroesophageal reflux disease
GI	Gastrointestinal
JBI	Joanna Briggs Institute
MDT	Multidisciplinary team
PRISMA	Preferred Reporting Items for Systematic Reviews and Meta-Analyses
PROSPERO	International Prospective Register of Systematic Reviews
RCT	Randomized controlled trial
SEMS	Self-expanding metal stent(s)
SPSS	Statistical Package for the Social Sciences
TACE	Transarterial chemoembolization
TEF	Tracheoesophageal fistula
TEVAR	Thoracic endovascular aortic repair
